# Professor H. R. Smith’s Valedictory Address

**Published:** 1854-04

**Authors:** 


### *Professor H. R. Smith's Valedictory Address.*

Ladies and Gentlemen,—

It has been the practice in this institution, ever since it was chartered, for some one of its professors to give a few words of parting advice to the graduating class at the annual commencement.

This pleasing duty, at the present time, devolves on me. Pleasing, that I may address such advice as the present occasion, and their after necessities may require.

But painful, that it dissolves our connexion as teacher and scholar.

We feel ourselves honored and encouraged on the present occasion, by the personal presence of ladies and gentlemen of this city, whose countenance and approbation, we are sure, would not be given to any but a good work.

Thus encouraged, we come with confidence—I might say, with pride—to speak of the institution which calls us thus together, and for the useful science of which it has labored with success.

It has been but a few years since the name of Dental College was known in the vocabulary of men.

From remote antiquity there have been colleges for other objects.

There have been colleges for cardinals, colleges for priests, colleges for literary and scientific pursuits, and medical colleges.

But it was reserved for the legislature of Maryland to be the first to issue a charter for a Dental College. Ohio followed the example, and showed their regard for the public welfare, by chartering this, the second Dental College known on the records of men.

The benefits of dental colleges have already been felt in almost every state in the Union, and even in foreign countries.

Graduates of this institution are yearly going forth into the fields of labor in different parts of the world; and with pride do we say it, that so far as has come to our knowledge, not a single instance of empirical practice has been chargeable on any graduate of this institution. The reason for this is plain. A course of studies here effects two important objects, wholly incompatible with professional quackery. In the first place, it imparts so much knowledge of the true principles of the art, that the causes of empiricism are entirely removed. In the second place, a regular course of instruction in this college, impresses the minds of the graduates so deeply with sentiments of scorn and contempt for all mal-practice, that great indeed must be the temptation that would entrance them in its guilt.

As the graduates of this college pursue their professional education, they learn the difference between honorable and dishonorable practice—the advantages of the one, and the disadvantages of the other.

They here learn to distinguish the true man of science from the boastful pretender.

Also, connected with their dental studies, they learn why such men as the Crawcours and Malans, and their imitators and rivals of less talent and enterprise, but of equal empiricism, attain neither abiding wealth nor honorable distinction, by their arts of imposture and knavery.

On the other hand, they see such men as Hale, Clark, Harris, Flagg, Maynard, Elliott, Westcot, and many others, who have obtained professional excellence which has given them rank among the most prosperous, useful, and esteemed men in our profession and community.

While they are taught to shun the practices of the first, they are also recommended to imitate the industry and virtue of the others.

Dentistry, in its very nature, is one of the learned professions. It has its science and its literature, its private instructions, and its public colleges; and men of sterling genius and learning, both in this country and Europe, have written largely and luminously, on all the branches of our art; so that all students and practitioners can have a voluminous library of dental books.

Dentistry is only a speciality of medicine and surgery; and our dental colleges recognize no one as fully competent for the dental profession, unless he is well skilled in general medicine and surgery.

Dentistry, though yet in its infancy, is claiming its place among the highest and most important of the literary and scientific professions. The motto is, onward and upward.

The public are learning the value of scientific and honorable operators, and the time may soon come when the quack and pretender will be known only as things that were. The dentist will go hand in hand with the family physician, and be equally as important to the comfort and happiness of mankind. Such is the object and aim of dental colleges; and such, we have reason to believe, will be the successful result.

Gentlemen—Graduates in Dental Surgery—

Time, in its rapid flight, has at length brought us to that period, when the mutual labors in which we have been so pleasantly engaged, have terminated.

The relation of Teacher and Pupil no longer exists between us—the time for our parting has arrived; and, in behalf of your preceptors, I bid you farewell.

But before I do this, and now that you are about to leave these halls to return to your homes, or to enter upon the arduous and responsible duties of the profession into which you have been admitted, I would embrace the opportunity to give you a few words of parting advice.

I shall not do this so much from any requirements or custom, as that, by admonishing you of some of the difficulties, disappointments, and vexations, that are more or less incident to the profession in which you are about to engage. The path, though strewn with many flowers to entice you to enter, has many secret thorns, which will annoy you, as you travel its mazy windings.

Of these, I would admonish you, that you may be the better prepared to encounter and overcome them.

There is no sweet without its bitter, and no happiness without its cares and pains.

True fame and distinction are only obtained through laborious study, patient and hard labor, cares, vexations, and responsibilities.

That your time has not been misspent in which you have been engaged in your preparatory studies, the credentials you hold in your hands are ample proof.

The ordeal which you have been long looking for with solicitude, you have passed in a manner highly creditable to yourselves, and gratifying to your teachers and friends.

You have now achieved the first object of your toil and ambition; and if, as I doubt not it is the next object you have in view, to gain the highest point of skill in your profession, let me, by way of encouragement, say, that success will be sure to crown your exertions.

All things are possible to him who willeth.

It is with yourselves whether you write your autographs on the tablet of the highest summit of professional excellencies, or let your names pass from time with no encomium or kind regret.

You are now about to enter upon the practice and duties of an important profession, which rests its claims to public favor on its general utility as a branch of the healing art.

And if you follow out the principles and practice here begun, you will be esteemed, honored, respected, and rewarded, as really and fully as members of any other department of the medical profession.

But, when you leave the library, the lecture room, the laboratory, and your teachers, do not, I pray you, consider your education as finished, or cease to study, and labor, in improving yourselves and the profession.

You are now about to go to your fields of labor, and, although you must look to your labor for a support, and reward for your toil, you must never forget that the field is one of humanity, requiring the utmost care and gentleness in alleviating pain and suffering, and the constant exercise of patience and forbearance with your willing, but timid, patrons. You will thus, by a deportment uniformly decorous and gentlemanly, meet with marked good-will, and secure from all, respect and kindly feeling. You will thus, not only secure friends, but retain them.

Gentlemen, it is your duty, not only to preserve your profession in the respectable condition in which you find it, but to labor hard to raise the standard of professional excellence.

This you owe to society, which has a right to expect every man to do his duty, in making society wiser and better—you owe it to your predecessors, who have labored long and hard in the cause—you owe it to yourselves, who must stand or fall by the issue; and you owe it to this institution, which has a claim on you as its graduates.

Wherever you go, then, let the dignity of your deportment, your manly self-confidence, your untiring assiduity in making good your profession, your stern integrity in doing justice to all men, your pure and uncontaminated morality, your gentlemanly habits in all respects, be your sure and successful passport to the favor of mankind and your profession.

With such credentials, you need not fear. Society will not be long in finding out the course you pursue, and will sooner or later reward you abundantly. Your exertions are sure to be crowned with success, and later generations honor your memory.

The dental profession, although at present filled by many able and efficient practitioners, is still a progressive one. Each year adds new jewels to the already glittering diadem of Dentistry. Like the ancient painters of Greece, who tried to paint the lineaments of the face of the beautiful Goddess who enchanted the world: one added a feature, another depicted an emotion of tenderness, each one trying to perfect the portrait, until nature herself was weary with the loving impress of mind on the lifeless and unthinking canvass.

Thus years of patient and unmitigated toil has been spent, without bringing to perfection that long-wished for object.

Thus it is with the temple of dental science; although, at present, comely and beautiful, still it is not yet finished. Each generation will add new ornaments to its already stately proportions.

The edifice is founded on a firm and permanent foundation, but it is for you and future generations to adorn, beautify, and honor. Cast your sacrifices before her altars, and then pay your vows and devotions.

One thing I would have you bear in mind, which is this: that no one can become a proper master of a profession who divides his time between it and another. There is an old maxim which is very aptly applied to those who are dividing their time among various pursuits, but are masters of none :— "A Jack at all trades, but good at none."

This saying, though homely, is nevertheless true. You will invariably find, that those who are trying to do more or less at sevaral trades or professions, are far from being good at either. But if you know of a man who has become eminent in any of the trades or professions, you will find that he has made some speciality his study, and by binding all his energies to that point, he is well instructed in all the windings and intricacies pertaining to it; and whatever book or instrument he touches, it is with a view to inform him or be useful to him in that particular branch to which he is devoting his time. To gain this end, it is necessary to call in more or less of the collateral sciences, to assist and explain the one we are pursuing. But the mark which you aim at must be kept constantly in view before you, and turn not until your object is accomplished.

In cultivating the various departments of knowledge in the profession into which you have entered — and which should enter into the education of every dentist who desires to become eminent — you will find ample scope for the exercise of the greatest energies of your mind.

And unless you thus keep them actively and constantly employed, you will be poorly prepared to meet the difficulties which will devolve upon you in your future career, and soon you will be left far, far behind in the race for the prize of professional excellence, while your more industrious competitors will have reached the goal, and received the reward due to persevering energy and industry.

This would be mortifying to yourselves, and painful to those friends who are looking anxiously for your honorable fame.

Strive, then, lest those who are less prepared than yourselves, by superior exertion, overreach you, and secure to themselves that which, by similar exertion, might be yours.

Eminence, fame, and success are sure to follow close in the rear of an honorable ambition and persevering energy, and before a determined will all obstacles flee away.

The indolent practitioner may well pause at the onset of his professional career, and calculate the dull chances of success.

As well might the ground-fowl think of reaching the eagle's dizzy hight in ærial space, as for the lazy and unambitious practitioner to think of reaching the lowest step of the temple of fame.

Let the dentist who is possessed of one sluggish feeling, retire from the profession, for dental science never yields a single laurel that is not earned by ardent toil, close study, and high enthusiasm.

Pursue not the dental profession, unless you do so with a full determination to surmount and overcome all difficulties, however imposing and fearful the front they may present. With a patience that never flags until perfection is gained, though the cases may be surrounded with perplexities and seeming impossibilities; with a warm and sympathizing heart, but a steady hand and cool judgment. All these are necessary to the dental practitioner who would unite moral worth, intellectual eminence, and surgical skill, in the elements of his professional character.

Gentlemen, in your endeavors, try to make every operation a successful one, and by so doing you will retrieve the errors of the ignorant, and remove a mass of prejudice from the public mind; and you will reflect both honor upon yourself and your scientific associates, and help to move forward the car of improvement in Dental Medicine.

By so doing, instead of being branded by the public as an impostor and knave, they will hail your approach as one "who comes to save—who touches to beautify."

Make nature your teacher, while, by the aid of science, you repair her decay, and heal her defects. Study the form of beauty in the human face, and when nature has ruined the proportions and symmetry, by a badly arranged or decayed denture, be thou ready to remedy the defects, and restore to primitive beauty and loveliness. Combine the scholar and the artist, and as fast as the mind conceives let the hand of art construct.

Let reason and conscience dictate, and the operation is sure not only to please your patrons, but bring honor and affluence to you as your reward, and while others are blessed, you will be abundantly rewarded.

In your practice you will have more or less opposition and competition, and at times have much to try your patience and urbanity. Others who are both destitute of skill and morals, will attack your reputation and art, but with a straightforward and upright course, you have nothing to fear. The weapons by which they hope to wound you, will rebound with double force on their own defenseless heads.

The laurels which they have gained by calumniating you will last but for a day, and the sunbeams of public favor, which have so illumined their path, will be lost to them forever, while full light, which first guides your steps, will grow brighter and brighter, until it burns with a steady and a brilliant blaze.

Spend not your valuable time in combating every little error and prejudice that exists, but rather win your way to public confidence by following the path of science and morality, thereby making a reputation for yourselves which calumny can never reach—one which time will only make brighter, and future generations honor and respect.

Such, and such only will be the reputation worth striving for.

Gentlemen, you now go out into the world, bearing in your own hands the diplomas of the Ohio College of Dental Surgery, as testimonials of your qualifications, but with them you have upon you vows, either expressed or understood, to do more worthily than those who have gone before you. You go out as alumni of an alma mater who has cared for you as children, and dismissed you with her blessing.

It is worth years of study and toil to leave a name on record in the archives of some hall of science — some dear place to be connected in the mind through all coming years of life, with early studies, blossoming hopes, and high aspirations of future usefulness.

You now have imposed on you a double duty, not only of working for your own emolument, but for your alma mater, and dental science in general.

The Ohio College of Dental Surgery now counts on you as its sons, and when the sun of its prosperity is rising in its full effulgency, to be among those who are proud to glory in its ascendancy, and help to bear the banner inscribed—" Excelsior."

But should fortune frown darkly, or withering blight fall heavily upon its prospects, it is for you with a giant's strength to lay hold of the great sources of public influence, and compel them to pay tribute to the institution which has given you the power to be a benefactor to your fellow man.

Thus will this institution gain strength from small beginnings —each year gaining friendship from each graduate it sends abroad.

And when, by a prosecution of your art, you have gained a reputation and fortune for yourselves, it is but right for us to expect you to exert your well-earned influence in society in favor of our infant college.

In this way, graduates of this institution who go out into the world, are like money loaned out at compound interest, the longer they are out, the greater the accumulation of both physical and intellectual strength, they return to their alma mater.

And, in after years, when your names are mentioned in connection with the Ohio Dental College, may the College say, with the proud Roman matron—"These are my jewels."

Apart from scientific qualification, no subject is of more importance to a dentist, than the strictest attention to his deportment and dress as a gentleman. Any one who expects to be useful or successful in his business, must not neglect his dress or manners; for few, very few indeed will be the applicants for dental aid, who will purchase exemption from pain, at the cost of loathing and disgust.

Ever cultivate a gentlemanly, engaging, sincere, and most respectful style of manners, both toward your patients and worthy professional brethren. As the most of your patients will be from the more cultivated walks of life, you should ever observe toward them a deportment of winning assiduity.

As much of the pain of your patients will be from nervousness and fear, your skill and kindness must ever be on the alert to mitigate and soothe.

Inspire a confidence in your patients in your sympathy and skill, and one-half of the pain you will inflict will be driven away by knowing that they have your sympathy, and that you will inflict no unnecessary pain.

Gentlemen, graduates in dentistry, you now go out into the world with a double resposibility resting on you, that of being useful and influential citizens, and skillful and confidential dental surgeons.

You go out into the world teeming with industry, competition, enterprise, and enthusiasm.

The mystery of the future enhances your position. The obscurity which rests over your fortunes makes the scene more interesting.

There is a pure fountain of sympathy which ever springs up among those who are associated together, even for a short time, for mental and physical improvement, and such ever feel a deep interest in each other's welfare. The exercise of this sympathy and feeling, I feel myself authorized to say, is as warm and intense in the bosoms of my associates as in my own.

And, in the name of the Faculty, I tender you their ardent wishes for your future success, usefulness, and honorable position in society, and fame in your professional career.

I would also express to you the satisfaction your general deportment, assiduity in study in the rudiments of our art, has given to us all, and that nothing of a serious nature has come up to mar the happiness of our present college connexion.

Go, then, gentlemen, the world is before you. The temple of professional fame, although situated on the summit of a steep and rugged mountain, can be reached.

The glittering crown is suspended on the pinnacle in your view, but it will take years of toil and perseverance to reach it.

No laurel is worth the wearing unless obtained by arduous toil, both physical and mental, and we prize the gem in proportion to the labor and cost in obtaining.

Set your standard of excellence high, and falter not in your exertions to attain it.

Maintain a high positition in professional etiquette, not only toward those in the dental profession, but those of the medical profession, remembering that dentistry is only a speciality of the great system of medicine and surgery.

In hours of leisure from your operations, make your books your constant companions, and then consult and advise with the eminent men who have practiced the art of dentistry before you.

Take them for your counsellors, and your way to success is certain.

Before many months, we hope to have the College whose graduates you are, sheltered beneath an edifice that shall be an honor to science and the far-famed "Queen City."

And then, on your anniversary visits, the recollection of your early visits will be transferred from these simple lecture-rooms to an edifice which, for centuries to come, shall bear the proud name of your alma mater—*The Ohio College of Dental Surgery.*

Here may we often meet to extend a brother's hand and welcome, and refresh ourselves on the subject of our favorite profession. Here may you listen to many valedictions; and when time, with his ruthless hand, has laid low the heads of your present instructors, may you still be mounting higher and higher in your profession.

Gentlemen graduates, in the name of the Faculty, I again bid you farewell, with our best wishes for your prosperity here, and happiness in the world to come.

